# A mobile sound localization setup

**DOI:** 10.1016/j.mex.2020.101131

**Published:** 2020-11-05

**Authors:** J.A. Wasmann, A.M. Janssen, M.J.H. Agterberg

**Affiliations:** a Department of Otorhinolaryngology, Donders Institute for Brain, Cognition and Behaviour, Radboud University Medical Centre, Nijmegen, the Netherlands; b Department of Biophysics, Donders Institute for Brain, Cognition and Behavior, Radboud University, Nijmegen, the Netherlands

**Keywords:** Sound localization, Binaural processing, Directional hearing, Remote hearing test

## Abstract

In this paper, a mobile sound localization setup is described that can be used to measure a persons’ localization performance in a sophisticated way. With this mobile setup, researchers can travel to subjects, and studies are not limited by the willingness of participants to visit the clinic. In the setup, sounds are presented within a partial sphere in both the horizontal (−70° to 70° azimuth) and vertical (−35° to 40° elevation) plane. Participants are asked to indicate the perceived sound origin by pointing with a head-mounted LED. Head movements are recorded and instantly visualized (i.e. online target response plots). Depending on the research question, the setup can be adjusted for more advanced or simplified measurements, making the setup suitable for a wide range of research questions. The rationale for building this mobile setup was to test horizontal sound localization abilities (binaural hearing) and vertical sound localization abilities (monaural hearing) of children and patients who were otherwise not accessible for testing. In this setup loudspeakers are not visible and subjects are asked to indicate the perceived sound direction by a natural head-pointing response towards the perceived location. An advantage of the implemented pointing-method is the playful manner in which children are tested. They are ‘shooting’ at the perceived sound target location with a head-mounted LED and have fun while performing the test.

• We present a mobile sound localization setup suitable for measuring horizontal and vertical sound localization in children and adult patients in the convenience of their own environment.

## Graphical abstract

**Figure:**
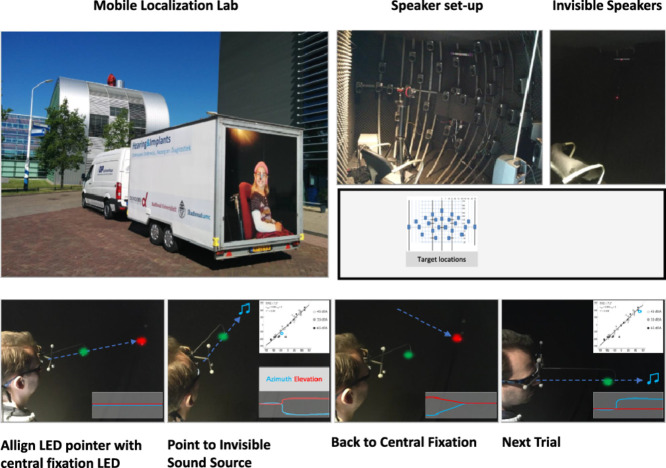

**Specifications Table**

**Table:** 

Subject Area:	Medicine and Dentistry
More specific subject area:	Audiology
Method name:	Sound Localization in a Mobile Laboratory

## Introduction

The ability to localize sounds is essential in daily life, for example, in traffic situations and for overall feelings of comfort [2,23]. A person with normal hearing is superb at localizing a sound's origin [12]. In the horizontal plane people use binaural processing of interaural differences in time (ITDs) and interaural differences in level (ILDs) to localize sounds, whereas in the vertical plane monaural spectral shape cues provide the necessary information to localize. In hearing impaired persons spatial hearing is compromised. By measuring sound localization capabilities, the inability to process monaural and binaural cues can be diagnosed and the effect of treatment can be assessed.

As sound localization is an important aspect of hearing, it has been investigated extensively in the past for normal hearing subjects and patients [3,^14^,20]. The presented mobile localization setup is designed to assess spatial hearing in both the horizontal and vertical plane using a head-pointing technique. The setup is preferred above other localization setups when:

i) There is a preference to test the participants’ localization abilities close to their home or school.
ii) A setup in a specific clinic is not suitable for measuring sound localization.

The initial reason for developing a mobile setup was to test normal-hearing children at schools. In the mobile setup, these children can be tested in a playful manner. The children are having fun while ‘shooting’ with their head-mounted pointer in the perceived sound direction. Testing at schools enables researchers to measure groups of children in a convenient way, without asking them and their parents to make a trip to the research institute, which could be a barrier to participate in studies.

An additional benefit of the setup's mobility is the opportunity to evaluate different treatment options for specific groups of patients at nearby clinics. This is of particular interest in Europe because due to the differences in reimbursement systems among countries a variety of different treatments are available for similar patient populations.

Because the mobile setup has proven to be a successful tool [17,^21^,22], we provide here essential details for others who consider building a localization setup. The paper describes the setup, the measurement procedures, the stimulus possibilities, the standard analysis and display of results, and we discuss the validity, applicability, and limitations of the mobile setup. We recommend the use of a setup in which speakers are not visible and where subjects are required to indicate the direction of the perceived sound by a natural head-pointing response.

## Hardware setup

**Fig. 1:**
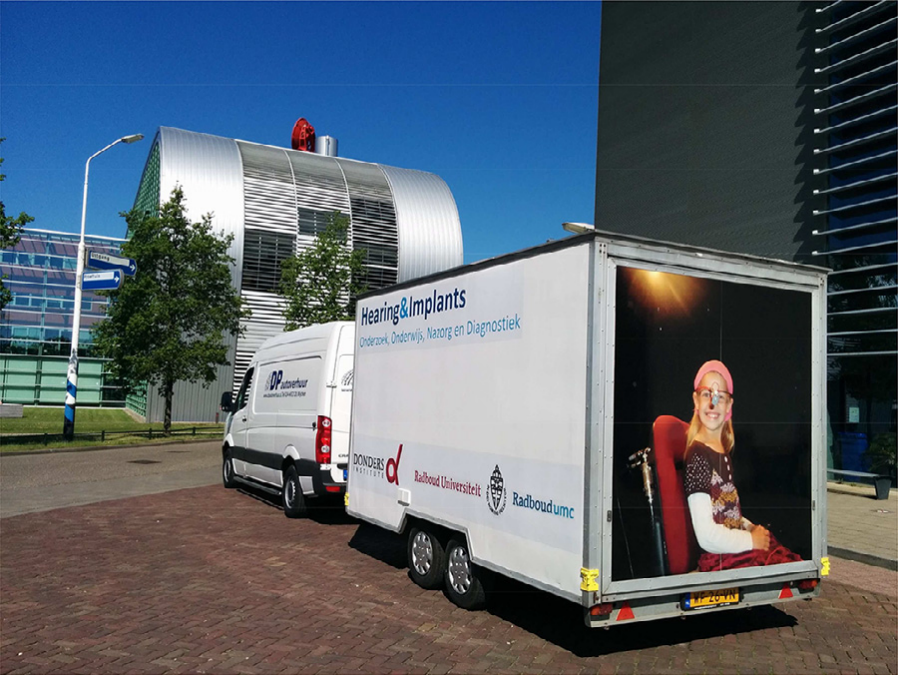
Mobile localization lab in a sound-isolated anechoic trailer.

**Fig. 2:**
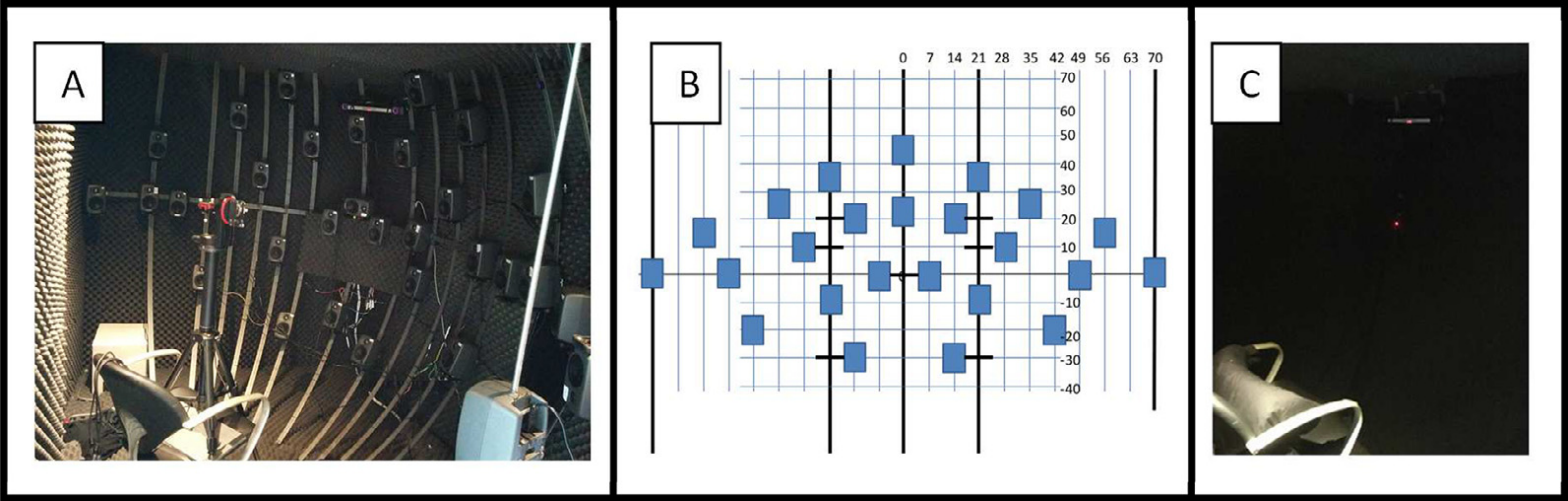
A.) The frame and position of the speakers relative to the chair. An additional speaker is mounted at +90 azimuth and 0° elevation (*bottom right corner*), which is not in the standard setup. B.) A schematic drawing of the speaker positions in the standard setup. C.) A photo of the setup with the speakers covered behind the sound-transmitting curtain.

Twenty-four speakers (Genelec 8010, 79 Hz–23 kHz, Genelec Oy, Iisalmi, Finland) are mounted on an iron framework built in a sound-isolated anechoic trailer (Fig. 1). Walls, ceiling and floor are covered with sound-attenuating foam (Fig. 2a). The speakers are positioned around the participant, at a constant distance of 1.2 m, within a range of +70° and −70° in the horizontal plane, and +40° to −30° in the vertical plane (Fig. 2), which makes it possible to assess both horizontal (azimuth) and vertical (elevation) sound localization performance. The standard distance between the speakers is 7° in the horizontal plane and 10°–20° in the vertical plane (Fig. 2B). Speakers can be repositioned if necessary. During standard experiments, a black sound-transmitting curtain is covering all speakers (Fig. 2C). Because speakers are not visible, visual and/or cognitive cues that likely affect the participant's response are not provided. The curtain covers approximately a range of + 90° to −90°, to create the illusion that a wider range is tested, similar to placing dummies speakers at the far left and right side of visible speaker array [5]. The curtain can be removed for experiments that require speaker visibility. Acoustic measurements (Clio fw Audiomatica, Firenze, Italy) at different positions in the room demonstrated slight reverberations only for low-frequencies (around 500 Hz). At the position of the subject's ears, the reverberation time is small (*T*_60_<0.09 s). The available lighting in the lab is dimmed during experiments to prevent any remaining visual cues. Performing the test under dimmed light conditions instead of complete darkness provides the ability to observe the participant, and is an advantage for testing patients with disturbed vestibular functioning [6].

**Fig. 3:**
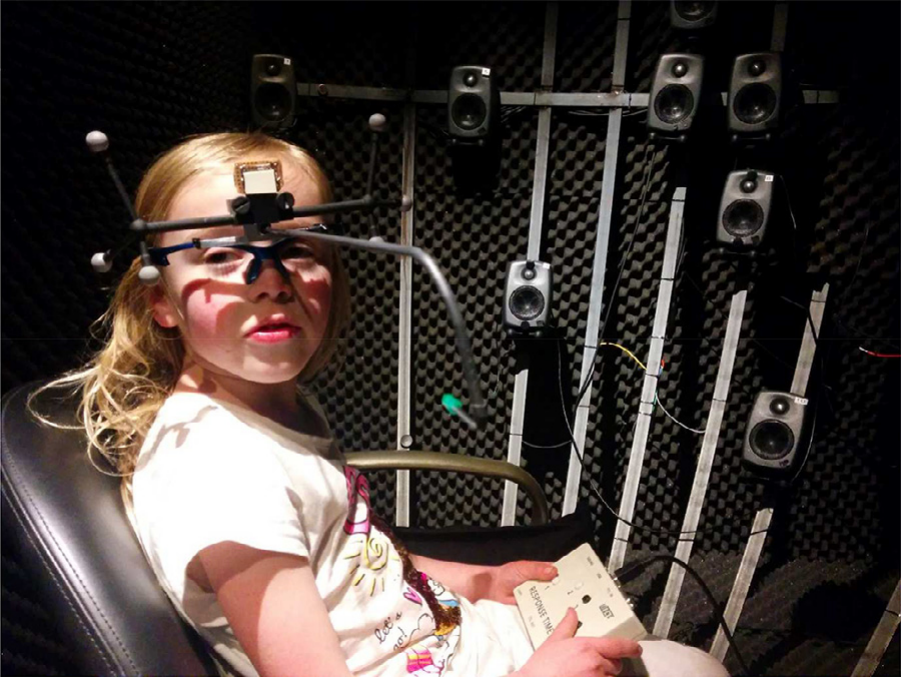
Frame with 3 infrared reflectors mounted on both sides and a green LED on a pointer centrally fixated in front of the participant's nose.

The participant is seated comfortably, while wearing a pair of custom-made glasses, which consists of a frame on which a rod with six infrared reflectors (Smarttrack, ART, Munich, Germany) is mounted. On the frame, also a green LED is centrally fixated in front of the participant's nose (Fig. 3). The participant indicates the origin of a sound by head-pointing, which has proven to be superior over other methods, like a joystick or touchpad, to indicate the perceived sound origin [4,14]. The chair can be adjusted in height to align the participant's ears with the central loudspeaker at azimuth 0° and elevation 0°. The participant is instructed to sit up straight during the experiment.

Matlab (The MathWorks, Natick, USA) is utilized to control the experiments via custom-made scripts in which the test settings can be adjusted. Stimuli are delivered by a sound card with 24 analog output channels (MOTU 24Ao, MOTU, Cambridge, USA) and an electronic board (Arduino Uno, Arduino, Somerville, USA), which triggers the fixation LED located at the center of the speaker array. For experiments that require more than twenty-four speakers, an additional sound card (MotuMini), and 8 Genelec speakers can be added. These additional speakers can also be used to widen the azimuth span. The costs for 24 active speakers is about $6000. The soundcard $1000 and the cables about $500. In addition, there are costs for the trailer and software licenses.

## Head pointing

The glasses are placed on the participant's face like normal glasses and stabilized with cotton straps to prevent movement of the glasses relative to the head. Before the start of the experiment, participants are instructed to move their head to test whether the glasses do not slip off. The glasses are constructed in a way that there is enough space around the ear to wear hearing aids or auditory implants, and can be used by persons with aural atresia. Head movements are recorded online via infrared cameras (Smarttrack, ART, Munich, Germany) tracking the position of the reflectors (Fig. 3). A red LED at 0° azimuth and 0° elevation serves as a central fixation light, which is at the level of the participant's ears.

The participant controls presentation of stimuli by a button box. After pressing the button, the central fixation LED is turned off, and a stimulus with 150 millisecond duration is presented after a variable 200–300 millisecond delay. The participant is instructed to point as fast and accurate as possible to the perceived sound origin. Each individual response (a completed head movement) is directly visualized online on a laptop in front of the researcher (*see 1.6 Data analysis and representation and Video 1*). The participant receives no visual reinforcement at the sound source. The researcher monitors whether head movements are made well within the acquisition time of 1.5 s. The acquisition time can be extended if needed. After the head movement, the fixation LED is turned on again (within 2 s), indicating the beginning of a new trial.

## Calibration and instruction

**Fig. 4:**
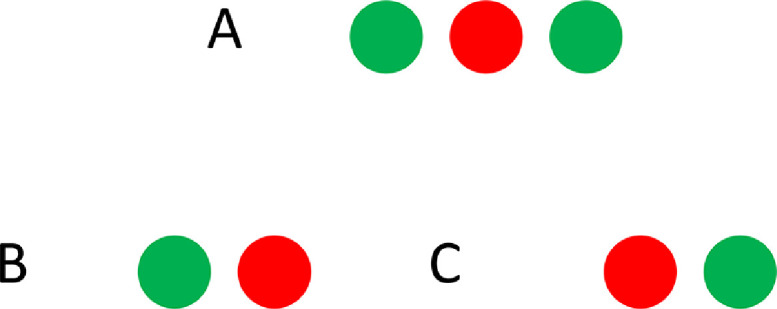
Schematic visualization of correct (*A*) and incorrect (*B and C*) alignment when fixating with the pointer (green) on the central LED (red). The green LED mounted to the pointing glasses is seen double (*A*) in case of correct alignment.

Before the actual start of an experiment, a calibration is performed. When a participant fixates on the central LED, azimuth should always be within a 2° deviation of zero. In order to verify this, the participant is instructed to look straight ahead and align the green LED on the pointer with the red fixation LED in the center of the speaker array. The participant should fixate on the red LED, and as a consequence, the green LED mounted to the pointer (Fig. 3) is seen double. The participant is instructed to align the double green LED with the red LED in the middle. A correct alignment is illustrated in Fig. 4A, incorrect fixation in Fig. 4B and C. After fixation, the participant has to make a head movement to the far right towards a visual target (LED) at azimuth +78° and elevation 0°. In case of more than 5° deviation in elevation the infrared reflectors (Fig. 3) on the frame are repositioned. The calibration procedure is repeated until the desired minimum offset is reached.

After calibration, the participant can be presented with a brief block of 12 broadband stimuli to get familiar with the test. The participant is instructed to fixate on the central red LED and push a button on the button box. After pushing the button, the red LED will turn off and the participant will hear a sound. The following instruction is given to the participant (for children more accessible language is used):

*‘Your task is to point with your nose as fast and accurately as possible towards the perceived sound direction. Please, localize with your whole head by pointing with the green LED pointer, do not only move your eyes, because we only measure your head movement. After you moved the green LED pointer, hold the pointer in that position for about 2 s, and then you can move back towards the position of the red fixation light. When the fixation light comes back on the next trial will start.*’

Usually, after 2 or 3 trials participants are instructed to initiate the next sound presentation by pressing the button themselves. During the training block, positive feedback like: *‘you are doing excellent’* or *‘perfect’* is provided. When the task is performed incorrectly the participant is instructed again. For instance, when the participant is still not pointing with the fixated LED to the target, but primarily looking to the target, the participant is asked to point to the experimenter's hand, and the difference between looking and pointing with the head-mounted LED is explained. This instruction helps especially when stimuli are presented about 70° opposite of the ‘frontal 0° direction’ of the participant. When needed, the participant is repeatedly instructed to look straight-ahead at the fixation LED before each trial.

## Stimuli

A standard experiment includes sound stimuli of broadband (0.1–20 kHz), low-pass (0.1–1.5 kHz) and high-pass (3 - 20 kHz) Gaussian noise bursts (duration of 150 milliseconds with 10 milliseconds on- and offset ramping). The low-pass and high-pass stimuli are applied to specifically measure the use of ITD's and ILD's. Stimuli are presented interleaved in a pseudorandom order with sound levels ranging from 45 to 65 dB (A-weighted = dBA, in 10 dBA increments) to limit the possibility that monaural level cues facilitate monaural localization abilities [19]. The on- and offset ramping prevents harmonic distortions that could provide an additional cue. If needed, the frequency band, sound level and number of stimuli can be adjusted within the Matlab code.

The stimulus duration is kept short to ensure the participant's head remains stationary during stimulus presentation, resulting in a true response to a single location. Sound presentations are evenly distributed over the two-dimensional frontal hemifield. Usually the minimum number of stimuli per condition is 75 (45 broadband stimuli at three different levels, 15 low-pass stimuli, and 15 high-pass stimuli) and can be changed depending on the research question. If it is preferable to present a low number of trials, for example because of a limited attention span, one can choose to only measure broadband stimuli to reduce testing time. Especially when working with children, the researcher should keep the number of stimuli to a minimum in order to reduce the risk of insufficient concentration and, consequently, less reliable responses. A trial refers to a single measurement and a block (of trials) refers to all trials that make up a complete experimental condition. The time required for one block varies from 5 to 10 min, depending on the number of stimuli (typically 45 broadband, 15 low-pass, and 15 high-pass stimuli) and the time the participant takes to initiate a new trial.

## Data analysis and representation

For each trial, the stimulus settings (coordinates azimuth and elevation, frequency, duration) and the raw data containing the recorded head movement (coordinates azimuth and elevation, response time) are stored in a data structure in the Matlab Workspace. The data structure is saved after each trial and can be used for offline analysis.

**Fig. 5:**
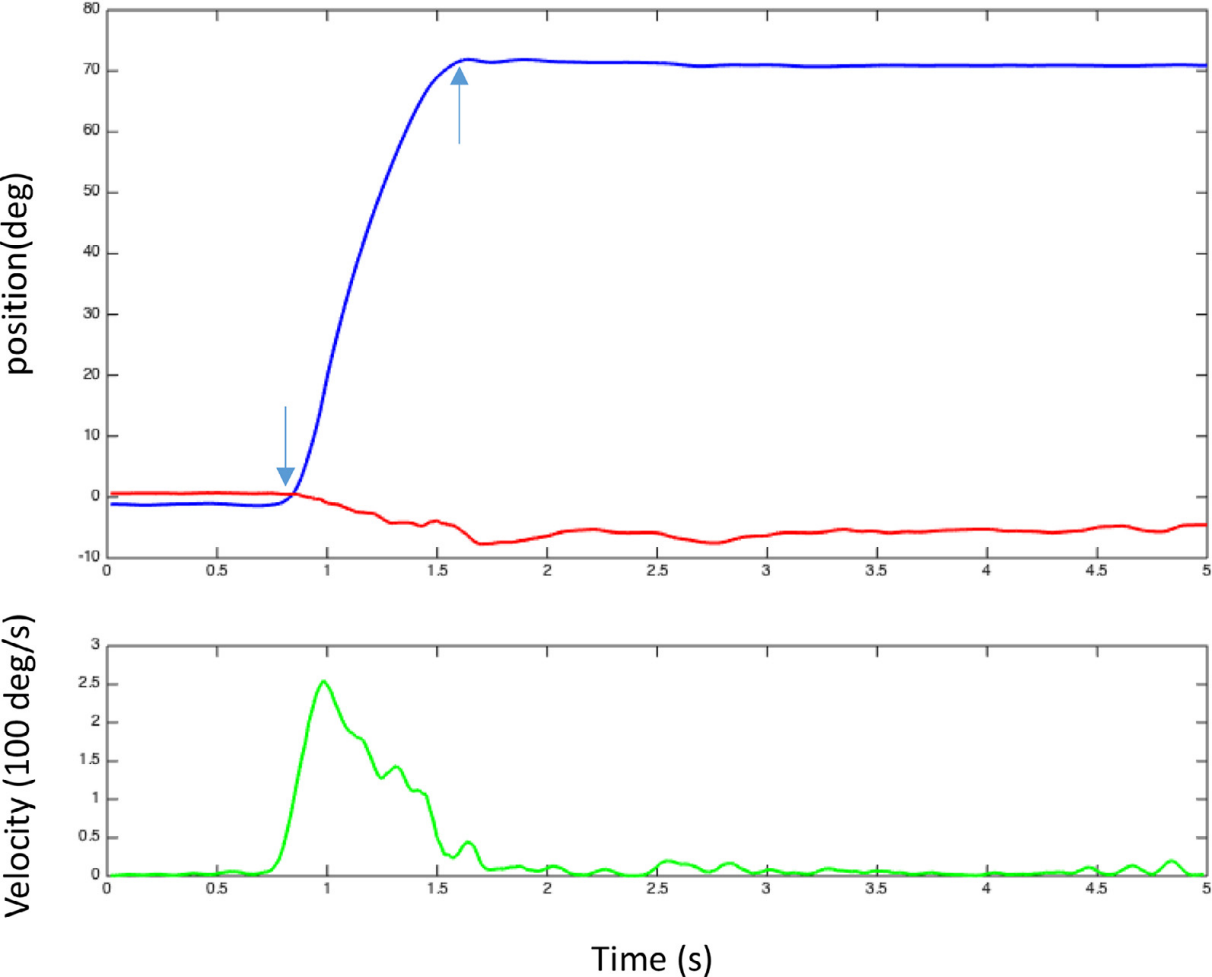
Online visualization of the head position (above) and head velocity (below) over time. The blue line indicates the azimuth position (in degrees) over time. The red line indicates the elevation (in degrees) over time. In both graphs the horizontal axis displays time in seconds. In the upper graph the vertical axis shows the head position in degrees. In the bottom graph the vertical axis expresses the velocity in 100° per second. The arrows indicate start- and end-points of the head movement.

During measurements, every individual response is visualized online by plotting the head movement over time (see video 1). Fig. 5 shows the head position (above) and head velocity (below). The initiation and end of the head movement is automatically detected online and can be manually corrected offline if needed.

**Fig. 6:**
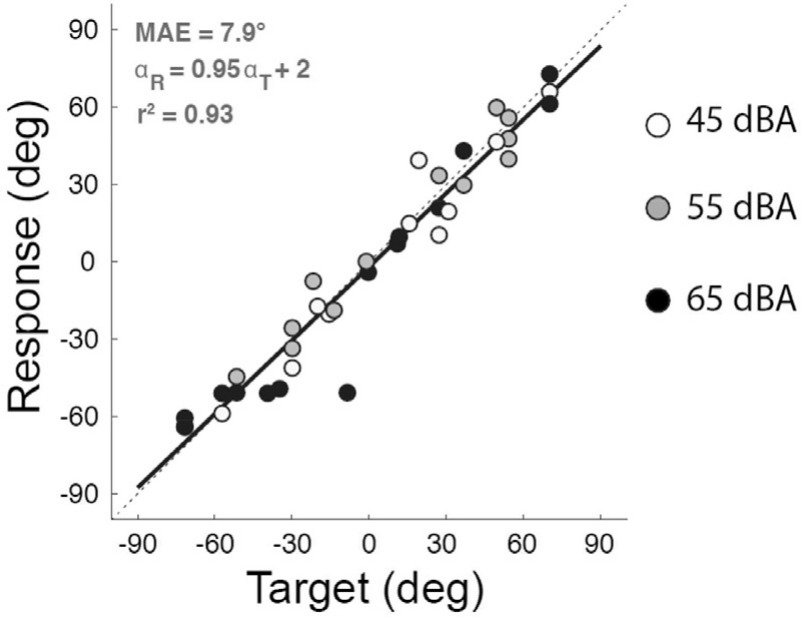
Target-response plot (Azimuth) of a representative normal hearing child. On the horizontal axis, the location of the target is projected, ranging from −90°, which is to the left of the subject, to +90°, which is to the right of the subject. On the vertical axis, the location to which the subject pointed is indicated. Also the vertical axis varies from −90° (response to the left) at the bottom to +90° at the top (response to the right). Each individual filled circle marks a response of the subject, and the presented sound level is indicated by the color (white: 45 dBA, gray: 55 dBA, black: 65 dBA). The dotted diagonal line illustrates perfect localization and the black line shows the best linear fit (Eq. (1)). The MAE expresses the deviation of the linear fit from the diagonal (Eq. (2)). *Adapted from “Improved directional hearing of children with congenital unilateral conductive hearing loss implanted with an active bone-conduction implant or an active middle ear implant,” by Vogt et al.*[21]*, Hearing Research, 370, p 241, Copyright 2018 by Elsevier.*

During each block of trials, preliminary target-response plots for azimuth and elevation are created online showing all completed trials. In Fig. 6, a target-response plot for a normal hearing child in the horizontal plane is shown, after offline corrections.

Afterward, all measurements are checked and erroneously processed start- and end-points of the head movement are manually corrected. Subsequently, a final target-response plot is created per block and a linear fit of the data is made based on below equation:

(1) $$\alpha_{RESP}=a\cdot \alpha_{TARG}+b\;\text{and}\;\varepsilon_{RESP}=c\cdot \varepsilon_{TARG}+d$$

In Eq. (1), $\alpha_{RESP}$ and $\varepsilon_{RESP}$ are the horizontal (azimuth) and vertical (elevation) response by the subject and $\alpha_{TARG}$ and $\varepsilon_{TARG}$ are target horizontal and vertical location, all expressed in degrees. Gain is defined by parameters *a* and *c*, for the horizontal and vertical plane respectively. Any bias in the responses is quantified by parameters *b* and *d*. In case of perfect localization the response of the listener is equal to the target, $\alpha_{RESP}=\alpha_{TARG}$ and $\varepsilon_{RESP}=\varepsilon_{TARG}$. This would mean the gains in the linear fits (a and c in Eq. (1)) are equal to 1 and the biases (*b* and *d* in Eq. (1)) are 0, and all responses fall on the diagonal.

The deviation from perfect localization is expressed by the mean absolute error (MAE), which is computed using below equation

(2) $$MAE=\frac{\sum_{i=1}^{n}\left|{\alpha^{i}}_{RESP}-\;{\alpha^{i}}_{TARG}\right|}{n}$$

The target-response plots are the standard way of presenting the data, of course, further analysis of the data is possible. For instance, in case of asymmetric hearing loss or unilaterally aided patients, Vogt et al. [22] calculated the gains, biases, and MAE for the stimuli presented to the left and right side separately. Furthermore, the response times can be analyzed, which could be a proxy for task difficulty.

## Method validation, limitations, and applications

The rationale for developing the mobile setup was to enable researchers to investigate sound localization in children and patients who were otherwise not accessible for testing. Vogt et al. [21] demonstrated the suitability of the mobile setup for testing children by measuring twenty-six normal-hearing children (Fig. 6). The sound localization assessment accuracy for this group was within 10° (average gain of 0.93 and a MAE of 7.3), and the testing time per condition per subject was shorter than 10 min. These results are comparable to earlier reports of localization performance in children [8,^13^,^14^,18]. The procedure proved to be rather intuitive and easy to understand, see videoclip 2 for a demonstration. Children are easily motivated to make fast head movements. This method is suitable to test the ability to process ITDs and ILDs (i.e. binaural hearing) because only auditory information is available and the response is not biased by cognitive factors [7].

**Fig. 7:**
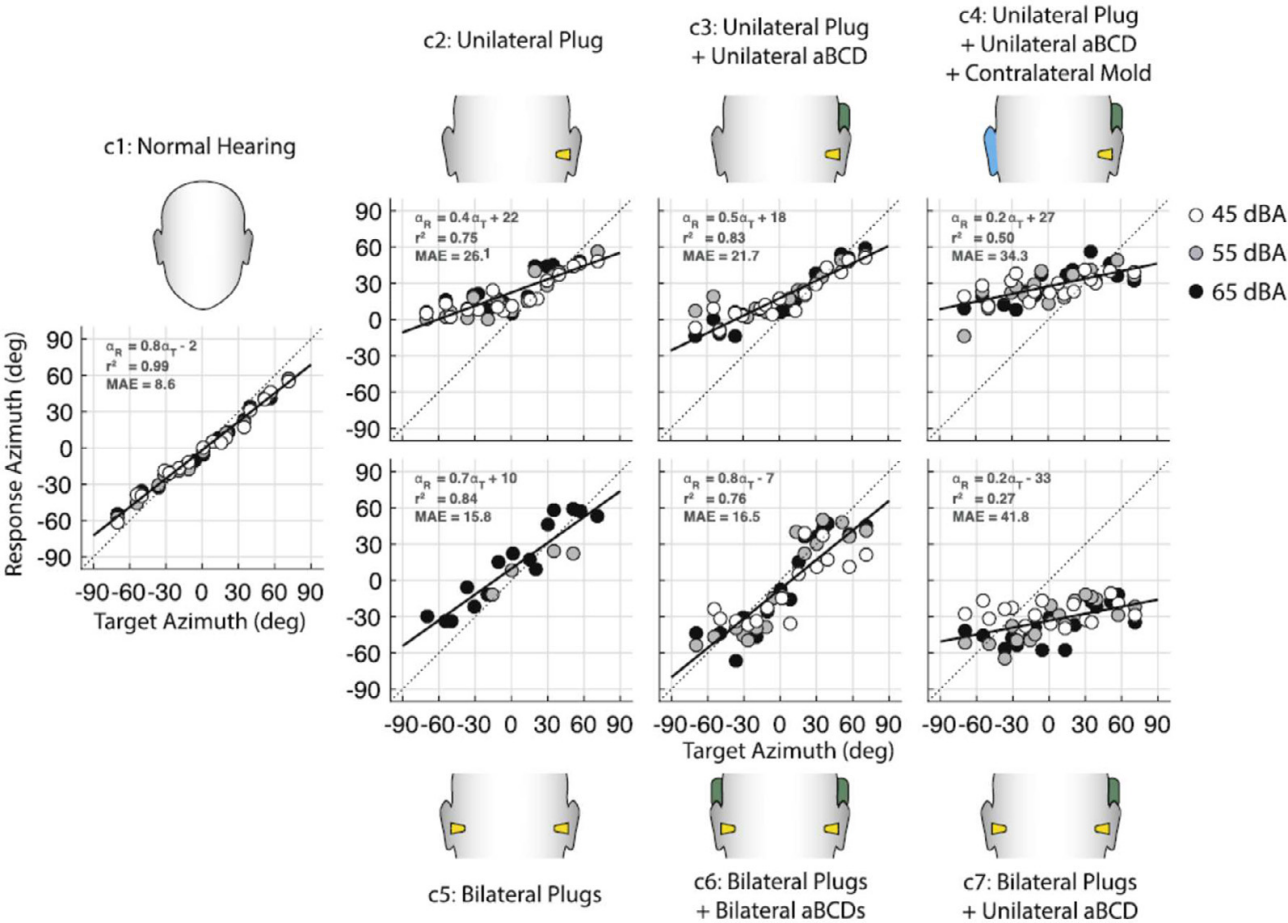
Overview of target-response plots (Azimuth) for seven different test conditions (C1–C7) of a representative normal hearing (plugged) hearing adult. The test conditions include normal hearing, plugged uni- and bilateral, and aided with a bone conduction device. *Reprinted from “Bilateral bone conduction stimulation provides reliable binaural cues for localization,” by*[17]*, Hearing Research, 388, p 4, Copyright 2019 by Elsevier.*

**Fig. 8:**
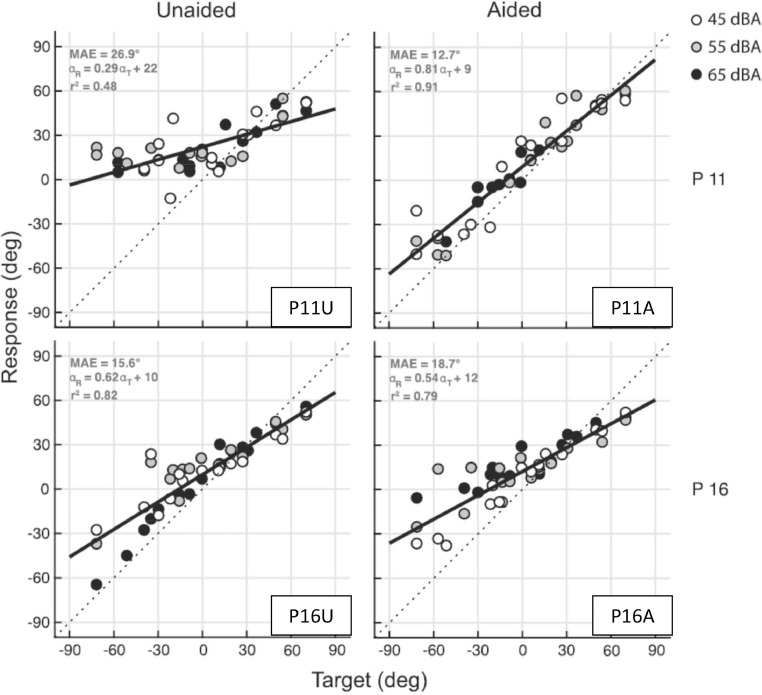
Target-response plots (Azimuth) of two children (P11 and P16) with severe unilateral conductive hearing loss on their left side. The two graphs on the left illustrate the unaided condition, whereas the graphs on the right show the children's performance using a bone conduction device. The sound levels are indicated with three different colors and calculation of MAE is done as in Eq. (2). *Adapted from “Contribution of spectral pinna cues for sound localization in children with congenital unilateral conductive hearing loss after hearing rehabilitation,” byVogt et al.*[22]*, Hearing Research, 385, p 4, Copyright 2019 by Elsevier.*

Recently studies have been published with normal hearing children tested in Kleef, Germany [21], unilateral hearing impaired patients tested in Lubeck, Germany [21], bilateral plugged normal hearing subjects in Nijmegen, The Netherlands ([17]; Fig. 7) and unilateral hearing impaired patients in Nijmegen, The Netherlands ([22]; Fig. 8). Because all subjects were tested in the same mobile setup, comparison of the data among studies is possible despite being performed in different European countries.

One of the outcomes of rehabilitation that can be tested is whether binaural hearing is restored. For example, in case of unilateral hearing loss or a large hearing asymmetry, accurate localization testing can provide information about the effectiveness of treatment. In the study presented by Vogt et al. [22], for two patients (P11 and P16 in Fig. 8), the large difference in localization behavior between the two patients in the unaided condition is striking. P11 unaided (P11U, Fig. 8) resembles the behavior of an acutely unilateral plugged normal hearing subject (C2, Fig. 7), whereas P16 does not show any difference between aided and unaided (P16U and P16A, Fig. 8). These major differences between patients demonstrate the importance of testing individual localization performance.

In addition to assessing the effects of rehabilitation with hearing aids or auditory implants, the setup is also suitable to study different specifications of hearing aids or auditory implants. For example, properties such as sound processing strategies, directionality of microphones, pre-processing (and time delay) in hearing aids or the effect of synchronization of bilateral worn devices can be investigated.

Another advantage of the described mobile lab is the possibility to investigate sound localization in the vertical plane. There is an increasing interest for testing sound localization abilities in single-sided deafness (SSD) patients using a cochlear implant [10,^9^,^11^,16]. It is known that patients with SSD can use monaural spectral pinna cues to localize sounds [1,^15^,19]. For this patient population, adequate assessment of unaided localization abilities is necessary to provide advice regarding the benefit of a cochlear implant.

Finally, there are a couple of drawbacks of the setup that should be mentioned. Firstly, a small but acceptable drawback of equipping a trailer with this setup is the limited sound isolation. For example, high-intensity external sounds, such as emergency helicopters or heavy rain, are audible. Secondly, the limitation to the size of the trailer (dimension 4.03 × 2.32 × 2.25 m) means a full circle for measuring in the horizontal plane does not fit. Therefore, front-back confusions cannot be tested. Thirdly, the range of elevation is reduced because of height constraints of the vehicle.

In summary, the presented mobile lab provides a versatile test environment to investigate horizontal and vertical sound localization abilities (i.e. binaural and monaural hearing) of both children and adults, normal hearing and hearing impaired, away from the clinic. Depending on the research question, adjustments can be made for more advanced or simplified measurements. The better our measurement methods become, the better we can assess the effectiveness of treatment.

## Declaration of Competing Interest

The authors declare that they have no known competing financial interests or personal relationships that could have appeared to influence the work reported in this paper.

## Appendix Supplementary materials

Image, video 1

Image, video 2

## References

[1] Agterberg M.J., Hol M.K., Van Wanrooij M.M., Van Opstal A.J., Snik A.F. (2014). Single-sided deafness and directional hearing: contribution of spectral cues and high-frequency hearing loss in the hearing ear. Front. Neurosci..

[2] Blauert J. (1997). Spatial Hearing: The Psychophysics of Human Sound Localization.

[3] Butler R.A. (1987). An analysis of the monaural displacement of sound in space. Percept. Psychophys..

[4] Goossens H.H., Van Opstal A.J. (1997). Human eye-head coordination in two dimensions under different sensorimotor conditions. Exp. Brain Res..

[5] Grantham D.W., Ashmead D.H., Ricketts T.A., Labadie R.F., Haynes D.S. (2007). Horizontal-plane localization of noise and speech signals by postlingually deafened adults fitted with bilateral cochlear implants. Ear Hear..

[6] Hartel B.P., Agterberg M.J.H., Snik A.F., Kunst H.P.M., van Opstal A.J., Bosman A.J., Pennings R.J.E. (2017). Hearing aid fitting for visual and hearing impaired patients with Usher syndrome type II a. Clin. Otolaryngol..

[7] Hofman P., Van Opstal A. (2003). Binaural weighting of pinna cues in human sound localization. Exp. Brain Res..

[8] Litovsky R.Y., Ehlers E., Hess C., Harris S. (2013). Reaching for sound measures: an ecologically valid estimate of spatial hearing in 2-3 year old children with bilateral cochlear implants. Otol. Neurotol.: Off. Publ. Am. Otol. Soc. Am. Neurotol. Soc. Eur. Acad. Otol. Neurotol..

[9] Liu J.F., Dai J.S., Wang N.Y. (2016). Effect of cochlear implantation on sound localization for patients with unilateral sensorineural hearing loss. Zhonghua Er Bi Yan Hou Tou Jing Wai Ke Za Zhi= Chin. J. Otorhinolaryngol. Head Neck Surg..

[10] Liu J., Zhou M., He X., Wang N. (2020). Single-sided deafness and unilateral auditory deprivation in children: current challenge of improving sound localization ability. J. Int. Med. Res..

[11] Lorens A., Kruszyńska M., Obrycka A., Skarzynski P.H., Wilson B., Skarzynski H. (2019). Binaural advantages in using a cochlear implant for adults with profound unilateral hearing loss. Acta Otolaryngol..

[12] Middlebrooks J.C. (2015). Sound localization.

[13] Nelissen R.C., Agterberg M.J.H., Hol M.K.S., Snik A.F.M. (2016). Three-year experience with the Sophono in children with congenital conductive unilateral hearing loss: tolerability, audiometry, and sound localization compared to a bone-anchored hearing aid. Eur. Arch. Otorhinolaryngol..

[14] Otte R.J., Agterberg M.J., Van Wanrooij M.M., Snik A.F., Van Opstal A.J. (2013). Age-related hearing loss and ear morphology affect vertical but not horizontal sound-localization performance. J. Assoc. Res. Otolaryngol..

[15] Slattery W.H., Middlebrooks J.C. (1994). Monaural sound localization: acute versus chronic unilateral impairment. Hear. Res..

[16] Snapp H.A., Ausili S.A. (2020). Hearing with One ear: consequences and treatments for profound unilateral hearing loss. J. Clin. Med..

[17] Snapp H., Vogt K., Agterberg M.J. (2020). Bilateral bone conduction stimulation provides reliable binaural cues for localization. Hear. Res..

[18] Van Deun L., Van Wieringen A., Van den Bogaert T., Scherf F., Offeciers F.E., Van de Heyning P.H., Desloovere C., Dhooge I.J., Deggouj N., De Raeve L. (2009). Sound localization, sound lateralization, and binaural masking level differences in young children with normal hearing. Ear Hear..

[19] Van Wanrooij M.M., Van Opstal A.J. (2004). Contribution of head shadow and pinna cues to chronic monaural sound localization. J. Neurosci..

[20] Vliegen J., Van Opstal A.J. (2004). The influence of duration and level on human sound localization. J. Acoust. Soc. Am..

[21] Vogt K., Frenzel H., Ausili S., Hollfelder D., Wollenberg B., Snik A., Agterberg M. (2018). Improved directional hearing of children with congenital unilateral conductive hearing loss implanted with an active bone-conduction implant or an active middle ear implant. Hear. Res..

[22] Vogt K., Wasmann J.A., Van Opstal A.J., Snik A.F.M., Agterberg M.J.H. (2020). Contribution of spectral pinna cues for sound localization in children with congenital unilateral conductive hearing loss after hearing rehabilitation. Hear. Res..

[23] Yin T.C., Smith P.H., Joris P.X. (2019). Neural mechanisms of binaural processing in the auditory brainstem. Compr. Physiol..

